# Parental mental health before and during pregnancy and offspring birth outcomes: A 20-year preconception cohort of maternal and paternal exposure

**DOI:** 10.1016/j.eclinm.2020.100564

**Published:** 2020-10-12

**Authors:** Elizabeth A Spry, Claire A Wilson, Melissa Middleton, Margarita Moreno-Betancur, Lex W Doyle, Louise M Howard, Anthony J Hannan, Mary E Wlodek, Jeanie LY Cheong, Lindsey A Hines, Carolyn Coffey, Stephanie Brown, Craig A Olsson, George C Patton

**Affiliations:** a Centre for Social and Early Emotional Development, School of Psychology, Faculty of Health, Deakin University, Geelong, Australia; b Murdoch Children's Research Institute, Melbourne, Australia; c Section of Women's Mental Health, Institute of Psychiatry, Psychology and Neuroscience, King's College London, London, United Kingdom; d South London and Maudsley NHS Foundation Trust, London, United Kingdom; e University of Melbourne, Melbourne, Australia; f Royal Women's Hospital, Melbourne, Australia; g Florey Institute of Neuroscience and Mental Health, University of Melbourne, Melbourne, Australia; h Population Health Science Institute, University of Bristol, Bristol, United Kingdom

**Keywords:** Preconception, Mental disorders, Paternal mental health, Birth outcomes, Cohort studies, Adolescent, Pregnancy, Small for gestational age, Preterm birth

## Abstract

**Background:**

Preterm birth (PTB) and small for gestational age (SGA) are increasingly prevalent, with major consequences for health and development into later life. There is emerging evidence that some risk processes begin before pregnancy. We report on associations between maternal and paternal common mental disorders (CMD) before and during pregnancy and offspring PTB and SGA.

**Methods:**

398 women with 609 infants and 267 men with 421 infants were assessed repeatedly for CMD symptoms before pregnancy between age 14 and 29 and during pregnancy. Associations between preconception and antenatal CMD symptoms and offspring gestational age/PTB and size for gestational age/SGA were estimated using linear and Poisson regression.

**Findings:**

In men, persistent preconception CMD across adolescence and young adulthood predicted offspring PTB after adjustment for ethnicity, education, BMI and adolescent substance use (adjusted RR 7·0, 95% CI 1·8,26·8), corresponding to a population attributable fraction of 31% of preterm births. In women, antenatal CMD symptoms predicted offspring PTB (adjusted RR 4·4, 95% CI 1·4,14·1). There was little evidence of associations with SGA.

**Interpretation:**

This first report of an association between paternal preconception mental health and offspring gestational age, while requiring replication in larger samples, complements earlier work on stress in animals, and further strengthens the case for expanding preconception mental health care to both men and women.

**Funding:**

National Health and Medical Research Council (Australia), Victorian Health Promotion Foundation, Australian Rotary Health, Colonial Foundation, Perpetual Trustees, Financial Markets Foundation for Children (Australia), Royal Children's Hospital Foundation, Murdoch Children's Research Institute, Australian Research Council.

**Research in context**

**Evidence before this study**

Consistent evidence links maternal antenatal symptoms of common mental disorders (CMD) with offspring preterm birth and small for gestational age. Although findings from animal studies implicate both maternal and paternal preconception stress in offspring birth outcomes, the extent to which maternal and paternal history of preconception CMD predict offspring preterm birth and small for gestational age in humans is unknown.

**Added value of this study**

In this population-based preconception cohort study, we prospectively assessed maternal and paternal CMD over 15 years from adolescence to young adulthood and in the third trimester of subsequent pregnancies. Our findings that up to 31% of preterm births may be attributable to processes relating to persistent preconception CMD symptoms in men heralds a paradigm shift for the developmental origins of health and disease. It highlights a need to further explore paternal CMD and other preconception risk factors as determinants of early life health, growth and development.

**Implications of all the available evidence**

Paternal mental health represents a neglected and potentially novel risk factor for preterm birth. While they require replication in other populations and a greater understanding of potential mechanisms, our findings support expansion of the focus of preconception care to include both men and women. Therefore new and potentially innovative approaches to preconception interventions will be necessary to optimise the health of future generations.

Alt-text: Unlabelled box

## 1 Introduction

Preterm birth (PTB) and small for gestational age (SGA) are leading causes of neonatal morbidity and mortality [1]. Early and mid-late PTB carry lifelong effects on health and development [2]. SGA is also associated with increased risk for neonatal morbidity and mortality, as well as short- and long-term adverse health outcomes and functional impairment, with these effects attributable primarily to fetal growth restriction [3]. Considerable research has focussed on antenatal risk factors for these adverse birth outcomes [4] but these only explain a small proportion of variance in PTB and SGA and preventive intervention efforts based on these risk factors are estimated to reduce PTB rates by only 5% [5]. For these reasons, understanding drivers of PTB and SGA remains a priority in public health policy and practice.

One explanation may be that some risks emerge well before pregnancy [6]. Maternal and paternal preconception exposures, including obesity, substance use, and education all predict patterns of offspring development [7]. In experimental animal research, parental preconception stress has been linked to offspring development via enduring effects on parental reproductive biology [8]. These findings point to an influence of preconception stress on offspring development even in the absence of antenatal exposure. There is some suggestion in humans that links between antenatal maternal mental disorder and offspring PTB may originate in the years immediately prior to pregnancy [9]. Associations between paternal preconception mental disorder and offspring later life mortality have been identified [10], but links to early offspring development, including birth outcomes, remain largely unstudied.

Mental health problems commonly become prominent in adolescence, persisting into adulthood and the transition to parenthood [11,12]. Investigating contributions of mental health problems, from adolescence to parenthood, to offspring birth outcomes may help elucidate mechanisms and identify possible intervention points. For example, the timing and persistence of any effects may implicate either sensitive periods or chronicity. Effects of exposure before, but not during, pregnancy may indicate direct preconception effects on parental reproductive biology, or mediation through other antenatal pathways [13]. Effects of parental mental disorder both before and during pregnancy may suggest effects of exposure at each phase via differing mechanisms, or confounding by underlying genetic or environmental influences of the association between mental disorder and offspring PTB or SGA [14]. Comparison of maternal and paternal associations is a further approach to investigating confounding effects of shared genes and environment, under the assumption that maternal and paternal exposures are similarly confounded [15]. For example, effects of maternal but not paternal symptoms may suggest a direct intrauterine effect rather than familial confounding [15,16].

Here, we used a two-generation cohort study with prospective data on maternal and paternal mental disorder over 15 years from adolescence to young adulthood, and again during pregnancy. We aimed to examine the association between maternal and paternal symptoms of common mental disorders (CMD) from adolescence to parenthood and offspring birth outcomes of PTB or SGA and whether these associations differ by timing of parental CMD symptoms and parent exposed.

## 2 Methods

### 2.1 Sample

The Victorian Intergenerational Health Cohort Study (VIHCS) is an ongoing prospective intergenerational study of preconception predictors of infant and child health, described elsewhere [17]. It arose from a cohort study commencing in 1992 in the state of Victoria, Australia (The Victorian Adolescent Health Cohort Study; VAHCS) [18]. Briefly, a representative sample of 1943 Victorian mid-secondary school students (1000 female) were selected via a two-stage cluster sampling design and assessed six-monthly during adolescence (VAHCS Waves 1–6: mean ages 14·9–17·4 years), and three times in young adulthood (VAHCS Waves 7–9: 20·7, 24·1 and 29·1 years). VIHCS began in 2006 during the ninth wave of VAHCS. Between 2006 and 2013 (participant age 29–35 years, encompassing median maternal and paternal ages for Australian births (Australian Bureau of Statistics, 2013)), VAHCS participants were screened at six-monthly intervals for pregnancies via SMS, email, and phone. Participants reporting a pregnancy or recently born infant were invited to participate in VIHCS, and asked to complete telephone interviews in trimester three, two months’ postpartum and one year postpartum for each infant born during VIHCS screening. Participants’ parents or guardians provided informed written consent at recruitment into VAHCS, and participants and their partners who participated provided informed verbal consent at subsequent waves.

### 2.2 Measures

#### 2.2.1 Exposures

*Preconception common mental disorder (CMD) symptoms* were assessed during VAHCS Waves 2–7 (participant ages 14–21 years) using the Revised Clinical Interview Schedule (CIS-R) [19]. a structured psychiatric interview designed to assess symptoms of anxiety and depression in community samples. The CIS-R has been validated for use with adolescent populations [20]. At each wave, total score was dichotomised at ≥12 to identify mixed depression-anxiety symptoms at a level lower than major depressive or anxiety disorder, but which a general practitioner would view as clinically significant [19]. At Waves 8 and 9 (participant ages 24 and 29), symptoms of psychological distress were assessed with the 12-item General Health Questionnaire (GHQ-12), a screening measure widely used to assess psychiatric illness in the general population. Total scores were dichotomised at ≥3, a threshold that indicates psychological distress with sensitivity 76% and specificity 83% [21,22], and corresponds to a CIS-R threshold of ≥12 [19]. In Wave 9, the Composite International Diagnostic Interview (CIDI) was used to assess depression (CIDI auto) [23] and anxiety (CIDI short form) [24]. Major depressive disorder and anxiety disorder were defined according to International Classification of Diseases, 10th revision (ICD-10). We constructed variables denoting presence of CMD symptoms at ≥1 adolescent wave (VAHCS Waves 2–6), and ≥1 young adult wave (VAHCS Waves 7–9). Continuity of CMD symptoms from adolescence to adulthood was defined as ‘none’, ‘adolescent only’, ‘young adult only’, and ‘both adolescent and young adult’.

*Antenatal CMD symptoms* were assessed in trimester three of pregnancy. Women were assessed using the Edinburgh Postnatal Depression Scale (EPDS) [25]. The EPDS is a 10-item rating scale designed to screen for postpartum depression and validated for antenatal use [26]. The total score (range 0–30) at each wave was dichotomised at a threshold (≥10) that is appropriate for use in community samples and when administered via telephone [27,28]. This cut-off has been recommended for use in detecting mild to severe postnatal depression, with a recent meta-analytic estimates of 95% sensitivity and 82% specificity in detecting major depressive disorder in the postpartum [29]. Men completed the General Health Questionnaire (GHQ-12), which was dichotomised at ≥3 as per the preconception young adult waves [19].

#### 2.2.2 Outcomes

Offspring sex, gestational age at birth, and birthweight were maternally reported (by VAHCS cohort participant or, for male VAHCS participants, by their partner) either at the first postpartum assessment at two months postpartum (86% of participants), at one year postpartum (3%), or during VAHCS wave 10 (11%; mean child age at assessment 3·8 years, SD = 1·9 years) [30]. Gestational age at birth was reported in completed weeks and categorised as preterm (PTB: <37 weeks’ gestation) and term (≥37 weeks’ gestation). Size for gestational age was calculated as birthweight z-scores, relative to the British Growth Reference [31,32]. These population norms provide expected distributions of birth weight according to gestational age and sex of infant. Small for gestational age (SGA) was defined as <10th percentile (birthweight z-score <1·28).

#### 2.2.3 Confounders

Our conceptual model included factors that were potential confounders of the associations between parental CMD symptoms and infant birth outcomes, excluding those potentially on the causal pathway from exposure to outcome [33]. Family of origin sociodemographic characteristics were: participants’ parents’ high school completion (neither v. at least one parent completed high school) and participants’ ethnicity (3-generation European descent). Measures of participant substance use at baseline in adolescence were: daily cigarette smoking (daily cigarette smoking at one or more waves v. no daily smoking), and binge drinking (ever v. never drank >5 drinks in a drinking occasion in the past week). Body mass index (BMI) was also considered as two binary variables of overweight (BMI ≥25 at any adolescent wave v. not overweight) and underweight (BMI <18.5 at any adolescent wave v. not underweight).

### 2.3 Statistical analysis

Data were analysed separately for women and men using Stata version 15 [34]. Multivariable robust Poisson and linear regression were employed for binary and continuous outcomes respectively, within a generalised estimating equation framework with robust standard errors, in particular to account for family clustering where more than one pregnancy per participant were included. The distribution of continuous outcomes was assessed for women and men using histograms stratified by exposure category, with the approximate symmetry deeming modelling of the mean via linear regression appropriate. Models were adjusted for parental ethnicity, BMI, adolescent smoking and drinking, and participant's parents’ education. In addition, the models with antenatal CMD symptoms as the exposure were adjusted for preconception CMD symptoms. Population attributable fractions were estimated for each level of preconception and antenatal CMD symptoms, using the adjusted risk ratios [35].

All analyses included participants who responded in at least one preconception wave and at least one perinatal wave. Multiple imputation by chained equations was implemented to handle missing data [36]. Datasets were imputed separately for women and men and for continuous and binary outcomes. 40 imputations were used according to proportion of participants (40%) with missing data on one or more variables [37]. All analysis variables were included in the imputation models, in addition to an auxiliary variable of divorce or separation of participant's parents by the end of wave six, which was predictive of non-response. Estimates were obtained by pooling results using Rubin's rules [38]. Available case analyses were also conducted in a sensitivity analysis (see supplementary material).

## 3 Role of the funding source

Funding sources had no role in study design, data collection, data analysis, data interpretation, or writing of the report. The corresponding author had full access to all data in the study and had responsibility for the final decision to submit for publication.

## 4 Ethics committee approval

Data collection protocols were approved by the human research ethics committee at the Royal Children's Hospital, Melbourne, Australia.

## 5 Results

**Fig. 1:**
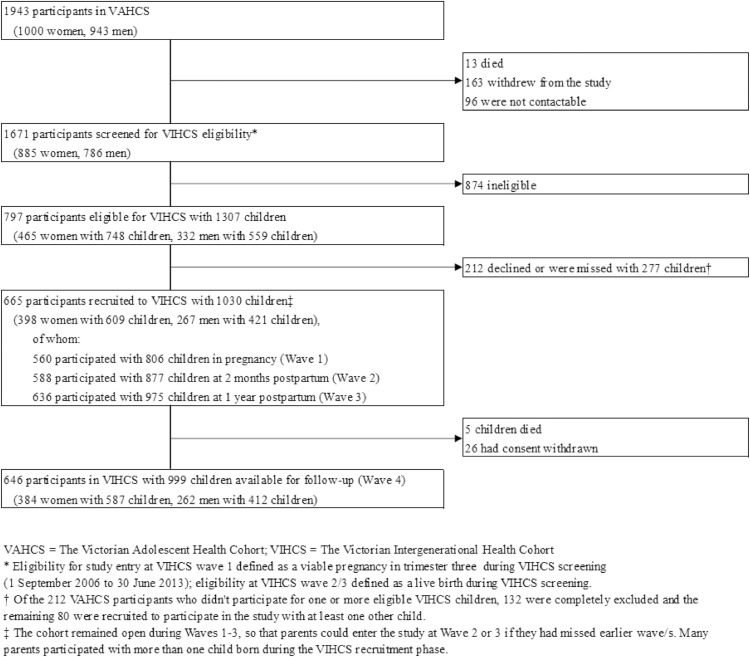
Sampling of the Victorian Intergenerational Health Cohort Study (VIHCS), from 2006 to 2014.

Our sample included 398 women with 609 children and 267 men with 421 children. The flow of participants through VIHCS is presented in Fig. 1. Demographics of those screened for, identified as eligible for and participating in VIHCS broadly matched those of the original adolescent cohort (VAHCS) [17]. Basic demographics of the sample and proportion of missing data in each variable are presented in Table 1. Approximately one half of women and one quarter of men had CMD symptoms in at least one of the adolescent study waves, with similar proportions seen during the young adult waves; 27% of women and 11% of men had CMD symptoms persisting across both adolescence and young adulthood, while 11% of both women and men had symptoms during pregnancy at 32 weeks gestation. Of those with complete birth outcome data, approximately 6% of both women and men had babies who were either preterm or SGA.

**Table 1:** Characteristics of the cohort (*N* = 1030), by sex of parent.

	Infants from women *N* = 609			Infants from men *N* = 421		
	n	%*	Missing (%)	n	%*	Missing (%)
**Parents of participant completing high school**						
Neither parent completed high school	222	36.8	6 (1%)	115	27.6	4 (1%)
At least one parent completed high school	381	63.2		302	72.4	
**Ethnicity (three generation European)**						
Three generation European	526	93.3	45 (7.4%)	366	96.3	41 (9.7%)
Three generation non European	38	6.7		14	3.7	
**Overweight in any adolescent wave (BMI >25)**						
Overweight	168	27.8	5 (0.8%)	85	20.4	5 (1.2%)
Not overweight	436	72.2		331	79.6	
**Underweight in any adolescent wave (BMI<18)**						
Underweight	94	15.6	5 (0.8%)	43	10.3	5 (1.2%)
Not underweight	510	84.4		373	89.7	
**Binge drinking in any adolescent wave**						
Binge dinking	191	32.1	14 (2.3%)	212	52.2	15 (3.6%)
No binge drinking	404	67.9		194	47.8	
**Cigarette smoking six or seven days per week in any adolescent wave**						
Smoking	121	19.9	0 (0%)	75	17.8	0 (0%)
No smoking	488	80.1		346	82.2	
**Symptoms of CMD in adolescence and young adulthood**						
None	236	38.9	3 (0.5%)	249	59.3	1 (0.2%)
Adolescent only	126	20.8		61	14.5	
Young adult only	82	13.5		65	15.5	
Adolescent and young adult	162	26.7		45	10.7	
Total number in adolescence	290	47.6		106	25.2	
Total number in young adulthood	244	40.1		110	26.1	
**Symptoms of CMD at 32 weeks gestation**						
No	371	88.8	191 (31.4%)	281	89.5	107 (25.4%)
Yes	47	11.2		33	10.5	
**Preterm birth (<37 weeks gestation)**						
No	565	93.2	3 (0.5%)	330	94.0	70 (16.6%)
Yes	41	6.8		21	6.0	
**Small for gestational age (< 10th centile)**						
No	566	94.5	10 (1.6%)	324	94.2	77 (18.3%)
Yes	33	5.5		20	5.8	
**Gestational age at birth (weeks)**⁎⁎	39.16 (1.84)			39.19 (1.96)		
**Size for gestational age (birthweight z-scores)**⁎⁎	0.27 (1.08)			0.20 (1.00)		

⁎ % among those with available data for that variable.

⁎⁎ Mean and standard deviation.

The unadjusted and adjusted associations between the two exposures of preconception and antenatal CMD symptoms and birth outcomes for women, using imputed data are shown in Table 2. There was evidence of an association between antenatal CMD symptoms and PTB, with maternal symptoms increasing PTB risk (relative risk (RR) 3·5; 95% confidence interval (CI) 1·3,9·1); this association remained after adjustment for the woman's parents’ high school completion, ethnicity, preconception CMD symptoms, overweight or underweight in adolescence and smoking and binge drinking in adolescence (adjusted RR 4·4; 95% CI 1·4,14·1). Assuming that this estimate reflects the magnitude of the true causal effect, this increased risk translates to a reduction in PTB by as much as 13% (the population attributable fraction), had no women experienced antenatal CMD symptoms. When gestational age at birth was considered as a continuous variable, this effect was in the same direction. Little evidence was found for an association between preconception or antenatal CMD symptoms and SGA, as either a binary or continuous outcome.

**Table 2:** Associations between preconception and antepartum symptoms of CMD with gestational age and size for gestational age in infants from women (*N* = 609).

	Preterm birth (<37 weeks gestation)									Gestational age at birth (weeks)								
	%⁎⁎	Unadjusted RR	(95% CI)		p-value	Adjusted RR*	(95% CI)		p-value	Mean (SD)	Unadjusted beta	(95% CI)		p-value	Adjusted beta*	(95% CI)		p-value
*Symptoms of CMD in adolescence and young adulthood*⁎⁎⁎																		
None	6.5									39.12 (2.09)								
Adolescent only	7.2	1.00	(0.33,	3.05)	0.998	0.94	(0.31,	2.89)	0.919	39.23 (1.66)	0.08	(−0.40,	0.55)	0.755	0.09	(−0.38,	0.57)	0.698
Young adulthood only	8.5	1.55	(0.51,	4.66)	0.437	1.60	(0.53,	4.79)	0.402	39.38 (1.89)	0.13	(−0.48,	0.75)	0.669	0.11	(−0.52,	0.73)	0.737
Both adolescent and young adulthood	6.2	1.02	(0.42,	2.49)	0.960	1.04	(0.40,	2.70)	0.931	39.07 (1.64)	−0.06	(−0.48,	0.35)	0.764	−0.08	(−0.51,	0.34)	0.697
*Symptoms of CMD at 32 weeks gestation*⁎⁎⁎⁎																		
No	5.1									39.20 (1.78)								
Yes	18.3	3.46	(1.31,	9.14)	0.012	4.42	(1.39,	14.06)	0.012	38.91 (2.15)	−0.25	(−0.97,	0.48)	0.507	−0.26	(−1.04,	0.51)	0.503

	Small for gestational age (<10th centile)									Size for gestational age (birthweight z-scores)								
	%⁎⁎	Unadjusted RR	(95% CI)		p-value	Adjsted RR*	(95% CI)		p-value	Mean (SD)	Unadjusted beta	(95% CI)		p-value	Adjusted beta*	(95% CI)		p-value
*Symptoms of CMD in adolescence and young adulthood*⁎⁎⁎																		
None	5									0.29 (1.10)								
Adolescent only	6.6	1.27	(0.45,	3.61)	0.647	1.34	(0.49,	3.64)	0.572	0.22 (1.04)	−0.09	(−0.40,	0.21)	0.547	−0.09	(−0.38,	0.21)	0.569
Young adulthood only	5.2	0.92	(0.27,	3.19)	0.896	1.17	(0.33,	4.07)	0.811	0.09 (1.10)	−0.14	(−0.48,	0.21)	0.435	−0.16	(−0.51,	0.19)	0.371
Both adolescent and young adulthood	5.6	1.08	(0.42,	2.76)	0.874	0.96	(0.38,	2.40)	0.925	0.34 (1.07)	0.01	(−0.24,	0.27)	0.922	0.07	(−0.19,	0.32)	0.606
*Symptoms of CMD at 32 weeks gestation*⁎⁎⁎⁎																		
No	5.2									0.26 (1.08)								
Yes	7.5	1.45	(0.55,	3.79)	0.452	1.82	(0.67,	4.95)	0.243	0.29 (1.05)	−0.01	(−0.29,	0.27)	0.947	−0.07	(−0.37,	0.22)	0.619

Imputed models using Poisson or linear regression within a generalised estimating equation framework with robust standard errors.

⁎ adjusted for parents of participant completing high school, ethnicity, overweight, underweight, binge drinking and tobacco smoking in adolescence. Analyses at 32 weeks gestation also adjusted for symptoms of CMD in adolescence and young adulthood.

⁎⁎ Proportion with outcome for each category of exposure using imputed data.

⁎⁎⁎ Preconception symptoms defined as: adolescent: CIS-*R* ≥ 12 in any wave 1–6; young adult: CIS-*R* ≥ 12 in wave 7 or GHQ-12≥3 in wave 8 or GHQ-12≥3 in wave 9 or CIDI diagnosis at wave 9.

⁎⁎⁎⁎ Antepartum symptoms defined as: EPDS≥10 at 32 weeks gestation.

In men (Table 3), there was evidence of an association between preconception CMD symptoms and PTB, with persistent CMD symptoms across adolescence and young adulthood showing the greatest magnitude (RR 8·8; 95% CI 2·4,32·2). This remained after adjustment (adjusted RR 7·0; 95% CI 1·8,26·8) and when gestational age at birth was considered as a continuous variable (unadjusted beta −0·8; 95% CI −1·5,−0·1 and adjusted beta −0·8; 95% CI −1·5,−0·01), translating to an earlier gestational age of around five days in men exposed to persistent preconception CMD symptoms compared with those without preconception symptoms. The corresponding population attributable fraction is 31%. There was little evidence of an association between paternal preconception or antenatal CMD symptoms and SGA and between paternal antenatal CMD symptoms and PTB, when considered as either a continuous z-score or binary outcome.

**Table 3:** Associations between preconception and antepartum symptoms of CMD with gestational age and size for gestational age in infants from men (*N* = 421).

	Preterm birth (<37 weeks gestation)									Gestational age at birth (weeks)								
	%⁎⁎	Unadjusted RR	(95% CI)		p-value	Adjusted RR*	(95% CI)		p-value	Mean (SD)	Unadjusted beta	(95% CI)		p-value	Adjusted beta*	(95% CI)		p-value
*Symptoms of CMD in adolescence and young adulthood*⁎⁎⁎																		
None	2									39.40 (1.50)								
Adolescent only	9.2	4.58	(0.97,	21.74)	0.055	4.28	(0.97,	18.92)	0.055	38.93 (2.73)	−0.47	(−1.26,	0.32)	0.246	−0.42	(−1.18,	0.33)	0.273
Young adulthood only	6.8	3.71	(0.81,	16.94)	0.090	3.71	(0.84,	16.35)	0.083	39.27 (1.93)	−0.18	(−0.87,	0.51)	0.608	−0.18	(−0.86,	0.51)	0.611
Both adolescent and young adulthood	17.2	8.79	(2.40,	32.17)	0.001	6.97	(1.81,	26.84)	0.005	38.54 (2.32)	−0.83	(−1.53,	−0.13)	0.020	−0.76	(−1.51,	−0.01)	0.048
*Symptoms of CMD at 32 weeks gestation*⁎⁎⁎⁎																		
No	5.7									39.23 (1.92)								
Yes	10.5	1.58	(0.34,	7.41)	0.563	1.19	(0.26,	5.41)	0.821	38.67 (2.22)	−0.53	(−1.42,	0.36)	0.244	−0.52	(−1.44,	0.39)	0.262

	Small for gestational age (<10th centile)									Size for gestational age (birthweight z-scores)								
	%⁎⁎	Unadjusted RR	(95% CI)		p-value	Adjusted RR*	(95% CI)		p-value	Mean (SD)	Unadjusted beta	(95% CI)		p-value	Adjusted beta*	(95% CI)		p-value
*Symptoms of CMD in adolescence and young adulthood*⁎⁎⁎																		
None	5.1									0.19 (0.93)								
Adolescent only	9	1.75	(0.55,	5.55)	0.338	1.45	(0.44,	4.72)	0.538	0.15 (1.10)	−0.05	(−0.41,	0.32)	0.809	−0.03	(−0.39,	0.33)	0.883
Young adulthood only	3.9	0.72	(0.11,	4.75)	0.731	0.68	(0.11,	4.34)	0.686	0.39 (0.98)	0.16	(−0.21,	0.53)	0.405	0.16	(−0.21,	0.52)	0.400
Both adolescent and young adulthood	9.6	1.81	(0.63,	5.19)	0.269	1.39	(0.46,	4.24)	0.560	0.07 (1.16)	−0.06	(−0.44,	0.32)	0.769	−0.02	(−0.39,	0.35)	0.927
*Symptoms of CMD at 32 weeks gestation*⁎⁎⁎⁎																		
No	6.1									0.18 (1.01)								
Yes	7.2	1.02	(0.24,	4.33)	0.984	0.96	(0.18,	5.02)	0.960	0.31 (1.03)	0.11	(−0.23,	0.45)	0.532	0.09	(−0.26,	0.44)	0.605

Imputed models using Poisson or linear regression within a generalised estimating equation framework with robust standard errors.

⁎ adjusted for parents of participant completing high school, ethnicity, overweight, underweight, binge drinking and tobacco smoking in adolescence. Analyses at 32 weeks gestation also adjusted for symptoms of CMD in adolescence and young adulthood.

⁎⁎ Proportion with outcome for each category of exposure.

⁎⁎⁎ Preconception symptoms defined as: adolescent: CIS-*R* ≥ 12 in any wave 1–6; young adult: CIS-*R* ≥ 12 in wave 7 or GHQ-12≥3 in wave 8 or GHQ-12≥3 in wave 9 or CIDI diagnosis at wave 9.

⁎⁎⁎⁎ Antepartum symptoms defined as: GHQ-12≥3 at 32 weeks gestation.

Available case analyses yielded a similar pattern of results for both women and men (see Supplementary Table 1 and Supplementary Table 2).

## 6 Discussion

Infants of men with persistent preconception CMD symptoms were more than six times more likely to be born preterm than infants of men without preconception CMD symptoms. Similarly, mean gestational age at birth among infants exposed to persistent paternal preconception CMD symptoms was almost one week earlier than among unexposed infants. If our estimates reflect the magnitude of the true causal effects, up to 31% of PTBs could be attributable to processes relating to persistent paternal preconception CMD symptoms. We also found an increased risk of PTB among infants exposed to maternal antenatal CMD symptoms, corresponding to a population attributable fraction of 13% of PTBs; these findings are consistent with earlier reports [39]. However, evidence of an association between paternal preconception CMD and offspring PTB has not previously been reported. These paternal associations persisted after adjustment for potential paternal socioeconomic, substance use, and BMI confounders. Thus this study provides a first indication that men's CMD symptoms in the decades prior to conception are associated with offspring PTB: an early life marker of disease and developmental risk.

The prevalence of preconception CMD symptoms in our study was similar to that previously reported in other prospective cohorts [40,41]. Likewise, the prevalence of antenatal CMD symptoms in women was similar to that of a previous meta-analysis [42], while the prevalence of antenatal CMD symptoms observed in men was at the upper end of previously reported meta-analytic bounds [43,44]. The prevalence of PTB and SGA were also broadly consistent with Australian population data [45].

Several mechanisms may explain the association between maternal antenatal CMD symptoms and offspring PTB. We accounted for a number of background factors and maternal characteristics that might increase risk of both maternal CMD symptoms and PTB, including family of origin demographics such as education and ethnicity, substance use, prior preconception CMD symptoms, and BMI. There remains potential for residual confounding, including lifetime experience of interpersonal violence or unplanned pregnancy [46,47]. There are also a number of potential mediators of these associations which warrant further investigation in larger samples. These include antidepressant use during pregnancy (although increased risk for PTB has also been found in those with untreated antenatal depression [39,48]), obstetric morbidities of preeclampsia, gestational diabetes, and gestational hypertension [49,50] and health risks such as substance use, poor nutrition and reduced engagement with antenatal care [2,51]. Antenatal CMD symptoms may also lead to adverse birth outcomes through impacts on maternal endocrine and immune functioning, all implicated in PTB [52,53].

We did not find evidence of preconception maternal effects. Prior findings are mixed; those studies reporting preconception associations have mostly assessed preconception stress exposure closer to the time of conception [9] so may better reflect antenatal effects. Evidence in this study for maternal antenatal associations but not maternal preconception or paternal antenatal, provide some further support for a potential causal effect of stress-related intrauterine processes.

There are similarly a range of processes by which paternal preconception CMD symptoms may be associated with offspring PTB. One possibility is residual confounding by early life characteristics, such as childhood maltreatment [54]. A further potential explanation is that the risk from paternal preconception CMD symptoms is mediated through maternal antenatal factors. Assortative mating is a tendency to choose partners with similar characteristics and has been observed in couples affected by CMD [55]. Partners of men with CMD are also at increased risk of exposure to other risk factors for adverse birth outcomes, including socioeconomic adversity, tobacco smoke exposure, and intimate partner violence [4,56,57].

Associations between persistent preconception CMD symptoms in men and infant PTB may also be mediated by paternal processes occurring in the months immediately before conception. In the testes, the production of new sperm cells from germ cells begins at puberty and continues throughout life. Production of each sperm cell takes around three months and is affected by environmental exposures, including substance use, poor nutrition, and exposure to environmental toxins, which are associated with both paternal CMD and offspring development [58]. Thus such stressors occurring during the months before conception may mediate associations between preconception CMD and offspring PTB [6].

Given the dose-response relationship between preconception CMD symptoms and offspring PTB, a further possibility is that chronic or recurrent CMD in the preconception years may have direct, cumulative and enduring effects on the male reproductive tract. There is some precedent for this proposal in the animal literature [13], although it has been little examined in humans. The pre-puberty period, with the formation of blood-testis and blood-epididymis barriers and commencement of spermatogenesis, has also been implicated as a sensitive window during which exposures may have a greater influence on reproductive development [59]. In our study, strong associations were observed between offspring PTB and paternal preconception but not antenatal CMD symptoms. These findings raise the possibility of sensitivity of the male germline to stress, not limited to the immediate preconception window but beginning in late childhood and extending into adolescence.

We did not find evidence of an association between preconception or antenatal CMD symptoms and offspring SGA in either women or men. These findings are consistent with results of prior meta-analyses [39,60]. One potential contributor is the biological heterogeneity of SGA, which comprises both constitutional differences attributable to genetic factors and processes relating to fetal growth restriction. Capturing these subtypes of SGA would require repeated assessments of fetal growth, beyond this study's scope. It remains possible that parental CMD may influence fetal growth restriction.

Strengths of this study include the repeated assessment of CMD symptoms across 15 years from adolescence to young adulthood and during subsequent pregnancies. Some limitations should also be noted. While we attempted to control for baseline confounders associated with both CMD and adverse birth outcomes, it is also possible that there is some residual confounding from unmeasured socio-environmental risks such as early life trauma, interpersonal violence, and socioeconomic adversity. Nonetheless, our finding of different maternal and paternal associations also suggest that results are not entirely attributable to familial confounding, under the assumption that maternal and paternal CMD symptoms are similarly confounded [16]. Infant birth outcomes were assessed by maternal report, which may be subject to reporting bias, although prior studies have found good agreement between maternal report and medical records of birthweight and gestational age at birth, particularly when continuous data are collected and when reported in the postpartum or early childhood [30,61,62]. In our study, most birth outcome data were collected at two months postpartum, with a smaller proportion collected at one year postpartum (3%) or the following VAHCS wave (11%, mean child age 3.8 years). This study was limited to infants born when parents were aged between 29 and 35 years. This design allowed us to maximise recruitment across the period of peak fertility in Australia but results may not be generalisable to older or younger parents with different risk profiles.

As with all cohort studies, the possibility of differential recruitment, attrition and non-response, and changing population demographics over time may have led to underrepresentation of some population groups. VAHCS maintains a high retention rate, and 85% of those with live births during screening participated in VIHCS. The retained and participating samples were broadly representative of the baseline VAHCS and eligible VIHCS samples respectively on measured baseline characteristics, but may differ on unmeasured confounders. Similarly, we were unable to examine associations within specific population groups known to have higher rates of preterm births, such as Aboriginal and Torres Strait Islander peoples [63,64], and this remains an area for future research. The sample in this study was relatively small, reducing precision of our estimates; findings should be interpreted as preliminary, and need replication in larger or pooled samples with similarly strong longitudinal designs. Missing data were low at each preconception and postpartum wave, but higher antenatally due to challenges of detecting all pregnancies before birth. We addressed potential biases arising from missing data using multiple imputation and the conclusions were unaltered.

Life course epidemiology and public health policy have long been grounded in assumptions around the causal primacy of maternal exposures and those occurring during pregnancy and early life [65]. While maternal antenatal risk factors for adverse birth outcomes remain an important focus for intervention, findings of the current study join a growing body of evidence highlighting the relevance of both paternal exposures and those prior to conception for offspring outcomes [6]. Given the high prevalence of CMD and the known adverse outcomes of PTB, the potential public health impact of their relationship is substantial. Our findings require replication in larger and diverse samples, with further examination of the mechanisms by which paternal persistent preconception CMD symptoms and maternal antenatal CMD symptoms might influence offspring PTB. Whether mediated by biological or psychosocial processes, our findings support calls for an expansion of preconception care to include strategies to address CMD, for both men and women [66]. The findings also underscore the importance of optimising mental health in adolescence and young adulthood, prior to pregnancy, emphasising the need for investment in coordinated care between child and adolescent, adult and specialist perinatal mental health services [67]. Intervention in adolescence to prevent onset and persistence of CMD into adulthood and pregnancy is likely to yield benefits not only for parents’ own continuing mental health, but also for the development of their children, either by preventing the potential direct impact of preconception CMD on offspring birth outcomes or interrupting potentially more complex developmental risk trajectories.

## 7 Funding

This work was supported by the 10.13039/501100000923Australian Research Council (DP180102447). Data collection for VIHCS was supported by the National Health and Medical Research Council (Australia), Australian Rotary Health, Colonial Foundation, Perpetual Trustees, Financial Markets Foundation for Children (Australia), Royal Children's Hospital Foundation and the Murdoch Children's Research Institute. CAW is supported by a 10.13039/501100000265Medical Research Council (UK) Clinical Research Training Fellowship (MR/P019293/1). GCP is supported by an NHMRC Senior Principal Research Fellowship (APP1117873) and Career Development Fellowship to JLYC (1141354). SB is supported by an NHMRC Senior Research Fellowship (APP1103976) and CAO is supported by an NHMRC Investigator Grant (APP1175086). MMB is the recipient of an Australian Research Council Discovery Early Career Award (project number DE190101326) funded by the Australian Government. LMH receives salary support from the South London and Maudsley NHS Foundation Trust and King's College London Biomedical Research Centre. AJH is supported by an NHMRC Principal Research Fellowship (APP1117148). LAH is supported by the Wellcome Trust (UK). Research at the Murdoch Children's Research Institute is supported by the Victorian Government's Operational Infrastructure Program.

## 8 Data sharing

Ethics approvals for this study do not permit the data to be made publicly available, due to limitations of participant consent and concerns regarding potential re-identifiability. Upon request, following publication of this article, the dataset subset used for these analyses can be made available to a named individual for the purpose of replication of research findings. The study protocol and variable documentation will also be provided. Data requestors will need to sign a data access agreement. Contact details for data requests can be found at https://www.mcri.edu.au/research/projects/2000-stories.

## Declaration of Competing Interest

LMH has received funding from NIHR HS&DR Programme for research into the effectiveness of perinatal mental health services in England.

## Appendix Supplementary materials

Image, application 1

## References

[1] Harrison M.S., Goldenberg R.L. (2016). Global burden of prematurity. Seminars Fetal Neonatal Med.

[2] Frey H.A., Klebanoff M.A. (2016). The epidemiology, etiology, and costs of preterm birth. Seminars Fetal Neonatal Med.

[3] Savchev S., Sanz-Cortes M., Cruz-Martinez R. (2013). Neurodevelopmental outcome of full-term small-for-gestational-age infants with normal placental function. Ultrasound Obstet Gynecol.

[4] Vogel J.P., Chawanpaiboon S., Moller A.B., Watananirun K., Bonet M., Lumbiganon P (2018). The global epidemiology of preterm birth. Best Pract Res Clin Obstet Gynaecol.

[5] Chang H.H., Larson J., Blencowe H. (2013). Preventing preterm births: analysis of trends and potential reductions with interventions in 39 countries with very high human development index. The Lancet.

[6] Stephenson J., Heslehurst N., Hall J. (2018). Before the beginning: nutrition and lifestyle in the preconception period and its importance for future health. Lancet.

[7] Patton G.C., Olsson C.A., Skirbekk V. (2018). Adolescence and the next generation. Nature.

[8] Keenan K., Hipwell A.E., Class Q.A., Mbayiwa K (2018). Extending the developmental origins of disease model: impact of preconception stress exposure on offspring neurodevelopment. Dev Psychobiol.

[9] Witt W.P., Wisk L.E., Cheng E.R., Hampton J.M., Hagen E.W (2012). Preconception Mental Health Predicts Pregnancy Complications and Adverse Birth Outcomes: a National Population-Based Study. Matern Child Health J.

[10] Costa D.L., Yetter N., DeSomer H (2018). Intergenerational transmission of paternal trauma among US Civil War ex-POWs. Proc Natl Acad Sci.

[11] Spry E., Giallo R., Moreno-Betancur M. (2018). Preconception prediction of expectant fathers’ mental health: a 20-year cohort study from adolescence. Br J Psychiatry Open.

[12] Patton G.C., Romaniuk H., Spry E. (2015). Prediction of perinatal depression from adolescence and before conception (VIHCS): 20-year prospective cohort study. Lancet.

[13] Toth M. (2015). Mechanisms of non-genetic inheritance and psychiatric disorders. Neuropsychopharmacology.

[14] Sheikh I.A., Ahmad E., Jamal M.S. (2016). Spontaneous preterm birth and single nucleotide gene polymorphisms: a recent update. BMC Genom.

[15] Smith G.D. (2008). Assessing intrauterine influences on offspring health outcomes: can epidemiological studies yield robust findings?. Basic Clin Pharmacol Toxicol Appl Pharmacol.

[16] Smith G.D., Lipsitch M., Tchetgen E.T., Cohen T (2012). Negative control exposures in epidemiologic studies. Epidemiology.

[17] Spry E., Olsson C.A., Hearps S.J.C. (2020). The Victorian Intergenerational Health Cohort Study (VIHCS): study design of a preconception cohort from parent adolescence to offspring childhood. Paediatr Perinat Epidemiol.

[18] Patton G.C., Coffey C., Romaniuk H. (2014). The prognosis of common mental disorders in adolescents: a 14-year prospective cohort study. The Lancet.

[19] Lewis G., Pelosi A.J., Araya R., Dunn G (1992). Measuring psychiatric disorder in the community: a standardized assessment for use by lay interviewers. Psychol Med.

[20] Patton G.C., Coffey C., Posterino M., Carlin J.B., Wolfe R., Bowes G (1999). A computerised screening instrument for adolescent depression: population-based validation and application. Soc Psychiatry Psychiatr Epidemiol.

[21] Goldberg D.P., Gater R., Sartorius N. (1997). The validity of two versions of the GHQ in the WHO study of mental illness in general health care. Psychol Med.

[22] Donath S. (2001). The validity of the 12-item General Health Questionnaire in Australia: a comparison between three scoring methods. Austr Psychiatry.

[23] Organization WH. CIDI-Auto version 2.1: administrator's guide and reference. 1997.

[24] Kessler R.C., Andrews G., Mroczek D., Ustun B., Wittchen HUJIjomipr. The world health organization composite international diagnostic interview short‐form (CIDI‐SF). 1998;7(4): 171–85.

[25] Cox J.L., Holden J.M., Sagovsky R (1987). Detection of postnatal depression. Development of the 10-item Edinburgh postnatal depression scale. Br J Psychiatry.

[26] Murray D., Cox J.L. (1990). Screening for depression during pregnancy with the edinburgh depression scale (EDDS). J Reprod Infant Psychol.

[27] Gibson J., McKenzie-McHarg K., Shakespeare J., Price J., Gray R (2009). A systematic review of studies validating the Edinburgh Postnatal Depression Scale in antepartum and postpartum women. Acta Psychiatr Scand.

[28] de Figueiredo F.P., Parada A.P., Cardoso V.C. (2015). Postpartum depression screening by telephone: a good alternative for public health and research. Arch Womens Ment Health.

[29] National-Collaborating-Centre-for-Mental-Health (2018). Antenatal and postnatal mental health: the NICE guideline on clinical management and service guidance. The British psychological society and the royal college of psychiatrists.

[30] Gartland D., Lansakara N., Flood M., Brown S.J (2012). Assessing obstetric risk factors for maternal morbidity: congruity between medical records and mothers' reports of obstetric exposures. Am J Obstet Gynecol.

[31] Cole T.J., Freeman J.V., Preece M.A.J (1998). British 1990 growth reference centiles for weight, height, body mass index and head circumference fitted by maximum penalized likelihood. Stat Med.

[32] Wright C., Booth I., Buckler J. (2002). Growth reference charts for use in the United Kingdom. Arch Dis Child.

[33] VanderWeele T.J. (2019). Principles of confounder selection. Eur J Epidemiol.

[34] StataCorp (2017). Stata statistical software: release 15.

[35] Rockhill B., Newman B., Weinberg C (1998). Use and misuse of population attributable fractions. Am J Public Health.

[36] White I.R., Royston P., Wood A.M (2011). Multiple imputation using chained equations: issues and guidance for practice. Stat Med.

[37] Bodner T.E. (2008). What improves with increased missing data imputations?. Struct Equ Model.

[38] Rubin D.B. (1987). Multiple imputation for nonresponse in surveys.

[39] Grote N.K., Bridge J.A., Gavin A.R., Melville J.L., Iyengar S., Katon W.J (2010). A meta-analysis of depression during pregnancy and the risk of preterm birth, low birth weight, and intrauterine growth restriction. Arch Gen Psychiatry.

[40] Moffitt T.E., Caspi A., Taylor A. (2010). How common are common mental disorders? Evidence that lifetime prevalence rates are doubled by prospective versus retrospective ascertainment. Psychol Med.

[41] Copeland W., Shanahan L., Costello E.J., Angold A (2011). Cumulative prevalence of psychiatric disorders by young adulthood: a prospective cohort analysis from the Great Smoky Mountains Study. J Am Acad Child Adolesc Psychiatry.

[42] Howard L.M., Molyneaux E., Dennis C.-.L., Rochat T., Stein A., Milgrom J (2014). Non-psychotic mental disorders in the perinatal period. Lancet.

[43] Paulson J.F., Bazemore S.D. (2010). Prenatal and postpartum depression in fathers and its association with maternal depression: a meta-analysis. JAMA.

[44] Cameron E.E., Sedov I.D., Tomfohr-Madsen L.M (2016). Prevalence of paternal depression in pregnancy and the postpartum: an updated meta-analysis. J Affect Disord.

[45] Li Z., McNally L., Hilder L., EA S (2011). Australia's Mothers and Babies 2009.

[46] Hill A., Pallitto C., McCleary‐Sills J., Garcia‐Moreno C (2016). A systematic review and meta-analysis of intimate partner violence during pregnancy and selected birth outcomes. Int J Gynecol Obstetr.

[47] Nesari M., Olson J.K., Vandermeer B., Slater L., Olson D.M (2018). Does a maternal history of abuse before pregnancy affect pregnancy outcomes? A systematic review with meta-analysis. BMC Pregnancy Childbirth.

[48] Jarde A., Morais M., Kingston D. (2016). Neonatal outcomes in women with untreated antenatal depression compared with women without depression: a systematic review and meta-analysis. JAMA Psychiatry.

[49] Andersson L., Sundström-Poromaa I., Wulff M., Åström M., Bixo M (2004). Implications of antenatal depression and anxiety for obstetric outcome. Obstetr Gynecol.

[50] Wilson C.A., Newham J., Rankin J. (2019). Is there an increased risk of perinatal mental disorder in women with gestational diabetes? A systematic review and meta‐analysis. Diabetic Med.

[51] Grote N.K., Bridge J.A., Gavin A.R., Melville J.L., Iyengar S., Katon W.J (2010). A meta-analysis of depression during pregnancy and the risk of preterm birth, low birth weight, and intrauterine growth restriction. Arch. Gen. Psychiatry.

[52] Christian L.M. (2012). Psychoneuroimmunology in pregnancy: immune pathways linking stress with maternal health, adverse birth outcomes, and fetal development. Neurosci Biobehav Rev.

[53] Gilles M., Otto H., Wolf I.A. (2018). Maternal hypothalamus-pituitary-adrenal (HPA) system activity and stress during pregnancy: effects on gestational age and infant's anthropometric measures at birth. Psychoneuroendocrinology.

[54] Vagero D., Rajaleid K. (2017). Does childhood trauma influence offspring's birth characteristics?. Int J Epidemiol.

[55] Nordsletten A.E., Larsson H., Crowley J.J., Almqvist C., Lichtenstein P., Mataix-Cols D (2016). Patterns of nonrandom mating within and across 11 major psychiatric disorders. JAMA Psychiatry.

[56] Ion R.C., Wills A.K., Bernal A.L (2015). Environmental tobacco smoke exposure in pregnancy is associated with earlier delivery and reduced birth weight. Reprod Sci.

[57] Oram S., Khalifeh H., Trevillion K., Feder G., Howard L (2014). Perpetration of intimate partner violence by people with mental illness. Eur J Public Health.

[58] Stuppia L., Franzago M., Ballerini P., Gatta V., Antonucci I (2015). Epigenetics and male reproduction: the consequences of paternal lifestyle on fertility, embryo development, and children lifetime health. Clin Epigenetics.

[59] Wu H., Hauser R., Krawetz S.A., Pilsner J.R (2015). Environmental susceptibility of the sperm epigenome during windows of male germ cell development. Curr Environ Health Rep.

[60] Littleton H.L., Breitkopf C.R., Berenson A.B (2007). Correlates of anxiety symptoms during pregnancy and association with perinatal outcomes: a meta-analysis. Am J Obstet Gynecol.

[61] Shenkin S.D., Zhang M.G., Der G., Mathur S., Mina T.H., Reynolds R.M (2017). Validity of recalled v. recorded birth weight: a systematic review and meta-analysis. J Dev Orig Health Dis.

[62] Olson J.E., Shu X.O., Ross J.A., Pendergrass T., Robison L.L (1997). Medical record validation of maternally reported birth characteristics and pregnancy-related events: a report from the Children's Cancer Group. Am J Epidemiol.

[63] Kildea S., Gao Y., Hickey S. (2019). Reducing preterm birth amongst Aboriginal and Torres Strait Islander babies: a prospective cohort study, Brisbane, Australia. EClinicalMedicine.

[64] Australian Institute of Health and Welfare (2017). Australia mothers and babies 2015 - perinatal dynamic data displays.

[65] Sharp G.C., Lawlor D.A., Richardson S.S (2018). It's the mother!: how assumptions about the causal primacy of maternal effects influence research on the developmental origins of health and disease. Soc Sci Med.

[66] Frey K.A., Navarro S.M., Kotelchuck M., Lu M.C (2008). The clinical content of preconception care: preconception care for men. Am J Obstet Gynecol.

[67] Public Health England (2018). Making the case for preconception care.

