# Supplementary material for: Parental mental health before and during pregnancy and offspring birth outcomes: A 20-year preconception cohort of maternal and paternal exposure
Source: eClinicalMedicine. 2020 Oct 12;27:100564. doi: 10.1016/j.eclinm.2020.100564 (PMC7599306; doi:10.1016/j.eclinm.2020.100564)
Supplement: Supplementary file 1 [file mmc1.docx]

Supplementary Table 1

Supplementary Table 2
